# A Qualitative and Quantitative Study Monitoring Airborne Fungal Flora in the Kidney Transplant Unit

**DOI:** 10.5812/numonthly.5379

**Published:** 2013-03-30

**Authors:** Mohammad Ali Afshari, Majid Riazipour, Reza Kachuei, Mojtaba Teimoori, Behzad Einollahi

**Affiliations:** 1 Applied Microbiology Research Center, Baqiyatallah University of Medical Sciences, Tehran, IR Iran; 2 Department of Medical Mycology, Faculty of Medical Sciences, Tarbiat Modares University, Tehran, IR Iran; 3 Department of Mycology and Parasitology, Medical Faculty, Baqiyatallah University of Medical Sciences, Tehran, IR Iran; 4 Molecular Biology Research Center, Baqyatallah University of Medical Sciences, Tehran, IR Iran; 5 Nephrology and Urology Research Center, Baqiyatallah University of Medical Sciences, Tehran, IR Iran

**Keywords:** Fungi, Renal Transplantation Unit Solution, Hospital

## Abstract

**Background:**

Solid organ transplantation patients are at high risk for opportunistic air-borne fungal infections due to using the potent immunosuppressive agents.

**Objectives:**

The current study aimed to qualitatively and quantitatively evaluate the fungal flora present in the air of Kidney transplant unit of Baqiyatallah hospital.

**Materials and Methods:**

In this prospective study, air samples from patient room, baths site, ICU and isolated room, corridor site and outside the ward were obtained by settled plate technique using plates containing Sabouraud's dextrose agar medium. In the current study, 36 agar plates containing Sabouraud dextrose agar medium were used. The plates were exposed for 20 min at height of 100-150 cm above the ground in units of hospital. Immediately after collection, samples were incubated at 27 ± 2ºC for four weeks. The slide culture method and Lacto-phenol cotton blue were used for definitive identification and staining fungal cultures, respectively.

**Results:**

The mean of colony forming units (CFUs) on indoor and outdoor plates was 6.6 ± 1.3 and 6 ± 1.9 / plate respectively. Statistical analysis showed that the observed difference is not significant. Also, the results showed that the mean of CFUs in the air of patient's rooms (6.8 ± 1.7), halls (4.5 ± 1.7), bathrooms (6.8 ± 1.5), and ICU rooms (3.2 ± 1.8) were not significantly different. The mean of different fungal genera isolated from indoor and outdoor plates were 1.9 ± 0.2 and 4 ± 0.5 genera/plate respectively, that indicates significant difference between indoor and outdoor air quality (P < 0.001).

**Conclusions:**

Lack of difference between quantity of outdoor and indoor air fungi indicates inefficiency of air control measures, and indoor lower genus diversity compared to outdoor air shows that there may be conditions that facilitate fungal growth in the environment of kidney transplantation unit.

## 1. Background 

Fungal infections may cause high morbidity and mortality in patients undergoing kidney transplantation (1-3). Although their spores are everywhere in the environments and apparently harmless to healthy persons, they can be fatal in patients taking the immunosuppressant drugs (4). With the application of more potent immunosuppressive agents, kidney recipients are increasingly more susceptible to a variety of atypical fungal infections with wide clinical presentations (5). Furthermore, hospital-acquired fungal infections can be encountered as life threatening opportunistic infections among organ transplanted patients (6). It is vital that where allograft is kept and operating halls do not contain airborne, opportunistic fungi (7-9); however, there are favorable conditions in hospitals for the growth of these fungal organisms (10).In half of organ recipients, infection occurs during the first year after transplantation (11, 12) and fungal infections are accounting for 11-15% of them (12, 13). Species of *Aspergillus* and *Candida* account for approximately 80% of fungal infections after solid organ transplantation (14). However, in a previous report from Iran, mucormycosis accounted for 52% of all invasive mycoses (2). Over the past two decades, the incidence of invasive aspergillosis among immunocompromised patients has progressively increased with a high mortality and morbidity rate (15). In addition, the incidence of post-transplantation of histoplasmosis was one case per 1000 person-years in large single-center series (16).

## 2. Objectives

The present study aimed to find genera and measure the level of airborne fungal contamination in kidney transplant unit of Baqiyatallah hospital.

## 3. Materials and Methods

### 3.1. Air Sampling and Mycological Examination

A prospective study was conducted to evaluate the airborne fungal contamination in Baqiyatallah kidney transplant center, Tehran, Iran. Air sampling at this unit was carried out between 25 June and 6 August 2008. Air samples from patient room, baths site, ICU and isolated room, corridor site and outside the ward were acquired by settled plate method using plates having Sabouraud's dextrose agar medium. Use of settle plate can provide a hint, whether an environment is more or less contaminated with airborne fungi. This technique is easy, frequently used, and sometimes preferred to other aerobiological samples (17, 18). In the current study, based on available space and room, 36 agar plates containing Sabouraud dextrose agar medium were used. The 90 mm diameter plates were exposed for 20 min at height of 100-150 cm above the ground in units of the hospital. The plates were then closed. Immediately after collection of samples, the petri plates were taken to the laboratory of mycology. These exposed plates were incubated at 27 ± 2˚C for four weeks. The number of colonies which appeared on the exposed plates were counted. Mycoflora was isolated and sub-cultured in the respective media for further identification. The slide culture method and Lacto-phenol cotton blue were used for definitive identification and staining fungal cultures, respectively. Microscopic examination of shape, size and arrangement pattern of spores and other vegetative structures of fungi were employed in their identification.

### 3.2. Statistical Approach

SPSS version 17.0 was employed to analyze the data. Quantitative variables were expressed as means ± SD, and results for the qualitative variables were expressed as frequencies and percentages. Average number of fungi isolated from different parts of the indoor air and outdoor air by the statistical methods (Kruskal–Wallis test and Mann–Whitney test), were compared and analyzed. All tests were two tailed, and Pvalues of less than 0.05 were considered statistically significant.

## 4. Results

**Figure 1.**
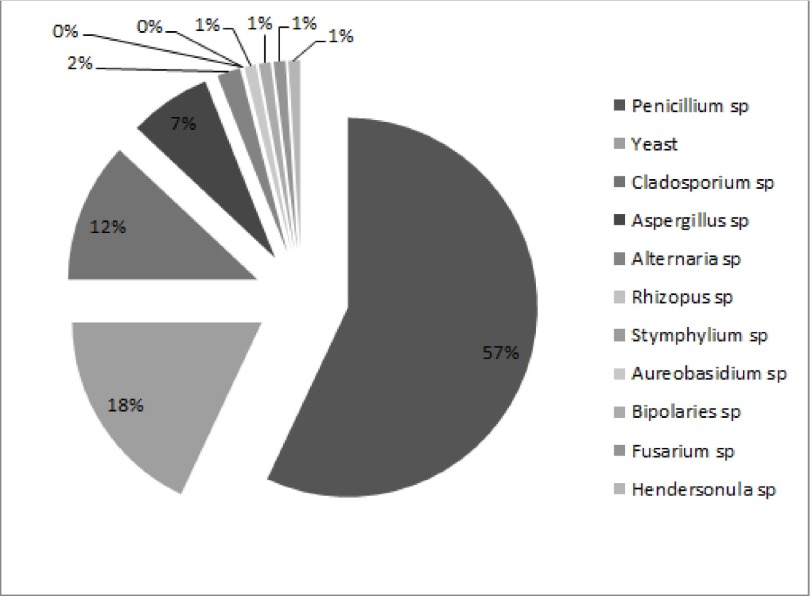
Disturbation of Fungal Isolated From in Ward and Out Ward

In this study 94% of plates were positive for fungal growth and a total of 220 colonies were isolated from 11 different types. The frequency of isolated fungi was *Penicillium spp.* (56%), Yeast species (18%), *Cladosporium spp.* (12%), *Aspergillus spp.* (7%), *Alternaria spp.* (4%), *Rhizopus spp.*, *Stemphylium spp*., *Aureobasidium spp.*, *Bipolaris spp.*, *Fusarium spp.* and *Hendersonula spp.* each (0.5%), respectively (Figure 1).

The number of colonies on the plates, outside and inside sites were 21% and 79% of the total number of colonies on the plate compared to the open air, 15% and 17% respectively. Average number of fungal colonies appeared on plates placed on the outside and inside. The average number of fungi isolated from air shows each destination. ANOVA showed that the average numbers of colonies grown on plates placed in different sites are not statistically significant (Table 1). When the samples were divided into two groups: external and internal, in the two groups, there were respectively 6.6 (3 ± 1.3) and 6 (± 0.9) colonies on plates (Table 2).

**Table 1.** Distribution of Fungi in Air Pollutants in Different Parts of the Kidney Transplant Hospital

Unit	No. of Plate	Colonies/Plate, mean ± SD	Genera/Plate, mean ± SD
**Corridor**	4	5.4 ± 0.9	2 ± 0.4
**Patient Room**	13	8.6 ± 1.7	8.1 ± 0.5
**ICU and Isolated Room**	4	2.3 ± 0.8	2 ± 0.4
**Baths**	8	8.6 ± 1.5	2 ± 0.3
**Outside the** **Ward**	7	6.6 ± 1.3	4 ± 0.5

**Table 2.** Distribution of Fungi in Air Pollutants in the Air, the Outer and Inner Kidney Transplant Hospital in Tehran

	No. of Plate	Colonies/Plate, mean ± SD	Genera/Plate, mean ± SD
**Outside the Ward**	7	6.6 ± 1.3	4 ± 0.5
**Inside the Ward**	29	6 ± 0.9	1.9 ± 2

Independent sample T tests also revealed no significant differences in the number of fungi in the air outside and inside. The difference between the average number of fungi on the plates placed in different sites was statistically significant. Average number of genera isolated in various parts of the ward did not show significant differences (P = 0.002). The average number of genera outside the ward (4 ± 0.5) with that of inside (1.9 ± 0.2) was different (P < 0.001) (Table 2). Significant difference (P < 0.001) was observed among the average number of genera outside the ward and Average number of genera in each of the inner sites of the ward, corridor (1.8 ± 0.5), patient room (4 ± 2), isolation room (4 ± 2) , ICU (1.8 ± 0.5) and bath (2 ± 0.3).

## 5. Discussion

The present study reported the distribution type and size of the fungal particles grew at 25. The major finding was that there were no significant differences among ward and out of ward colonies, which indicated the absence of a sufficient controlling system. Fungi are ubiquitous and can utilize many different substances for growth. Most fungal growth in domestic environments is accompanied by local humidity (19).The effect of the outdoor fungal flora on the indoor has been known; but, it is a proven fact that the indoor airborne fungi, regardless of the type come from two sources: the outdoor air and the indoor fungal colonization. Such colonization originates mainly in every wet, dark and poorly ventilated area (20). The majority of the indoor airborne fungal population comes from outdoor bases, in particular from the regional vegetation, which is identified to powerfully affect the nearby airborne fungal concentration. The infection with fungal spores in the lungs and their effect on human health hang on their virulence, genera and species, concentrations, and sizes (21). Our fungal genera distribution was similar to other studies, for example mallea reported that like (22). It has been shown that numerous proportions of hospital infections are initiated by fungi, such as *Candida albicans* and various species of *Aspergillus, Cladosporium,* and *Penicillium* (23-25), especially in the area of our interest. After solid organ transplantation, 80% of fungal contaminations include *Aspergillus* and *Candida* species (14). Among immunosuppressed patients, invasive *Aspergillosis*, the incidence of which has progressively amplified throughout the past 2 decades (15), leaves a severe complication and is very dangerous. Kanny et al. (26) have reported the most common genus was *Cladosporium*, followed by *Aspergillus, Penicillium* and Alternariaout door (27). Centeno showed that more frequent filamentous fungi were *Aspergillus, Penicillium* and *Fusarium* species. The isolated species with more frequency were *Aspergillus niger*, *Aspergillus flavus* and *Fusarium solani* (28). Martins showed that the most frequently isolated genera were *Cladophialophora*, *Fusarium*, *Penicillium, Chrysosporium* and *Aspergillus*. In their study, Yeasts found in nearly 40 percent of samples were from healthcare staff and more than 40 percent of furniture, with a majority of the genus *Candida*, followed by *Trichosporon* (29). Indoor fungi are a combination of those entered from outdoors and the ones which willingly grow and multiply indoors (30, 31). The indoor air fungal flora can vary from outdoor air equally quantitatively and qualitatively. The fraction of indoor to outdoor concentration of spores is usually less than one and is of alarm when this proportion is less. The internal sources of fungi modify the composition of indoor airborne fungi compared to outdoor air (32). As also seen in the current study this ratio was less than 1 but genera was different which shows some good place for some genre growths that must be considered. The fungal spore amounts of outdoor and indoor air vary significantly depending on various environmental and other issues (33). In the current investigation, the major genera in the kidney transplant unit were *Penicillium, Cladosporium*, *Aspergillus* and *Alternaria.*

Several studies have stated that *Cladosporium* is the most abundant genus identified from both indoor and outdoor samples (34-37). All fungal species were found significantly higher in bathes and patients rooms than in ICU and corridors in the current study. Unlu et al. (38) showed that fungal growth favors humid homes with high humidity levels and cold surfaces onto which moisture can condense. Therefore damp basements or humid bathrooms within an otherwise dry house can generate and spread mold spores throughout the hospital. Studies in Melbourne, Australia, found that mold levels were decreased in rooms with decreased dampness, which were frequently vacuumed (39). It is advisable that strict measures should be put in place to check the increasing microbial load in the hospital environment. It is necessary to use antiseptic air systems, ventilation systems, blocking the windows and installing air filters because the pulled air entrance. Control entry and exit doors and shutters to reduce unnecessary use of pots of flowers and plants applied to reduce spores. Indoor mold exposure occurs through infiltration of spores from outdoors and through growth of mold indoors. Decline strategies need to consider both sources of contamination. The mainstay of mold control is to decrease humidity through air conditioning, cooling, and closing of doors.
